# Enterocutaneous fistula occurring 10 years after an open umbilical hernia repair with placement of an onlay polypropylene mesh: A case report

**DOI:** 10.1016/j.ijscr.2020.02.004

**Published:** 2020-02-06

**Authors:** Mohammed Alshamali, Sana Sallam, Dhari Alzaid, Joud Abdulraheem, Khaleel Mohammad

**Affiliations:** Department of Surgery, Al-Adan Hospital, Ministry of Health, Kuwait

**Keywords:** Umbilical, Ventral, Hernia, Mesh, Fistula, Enterocutaneous

## Abstract

**Introduction:**

Ventral hernia repair is a common surgical procedure performed within the specialty of general surgery. Short and long term complications can arise after this procedure. Although rare, an enterocutaneous fistula may occur, leading to a significant morbidity and the possible need for surgical intervention.

**Presentation of case:**

We present a rare case of a 76 years old female, who presented with the sudden occurrence of an enterocutaneous fistula arising ten years after a primary umbilical hernia repair with placement of a polypropylene onlay mesh. She was also found to have a large recurrent umbilical hernia. She underwent a laparotomy with the identification of a mesh eroding into the small bowel lumen, causing an enterocutaneous fistula. An enterectomy was performed to remove the fistula with the mesh, and a small bowel anastomosis was created.

**Discussion:**

Ventral hernia recurrence is associated with risk factors including old age, obesity, wound infection as well as the type and location of the mesh used. As in this case, enterocutaneous fistula after hernia repair can occur due to multiple factors including mesh migration and erosion into near-by structures including bowel. These risk factors can perhaps be modified to possibly reduce the incidence of complications like an enterocutaneous fistula.

**Conclusion:**

The case highlights a rare but serious complication associated with a ventral hernia repair. It also addresses key aspects with regards to the possible mechanisms involved in the occurrence of an enterocutaneous fistula following a hernia repair with the use of a synthetic mesh.

## Highlights

• An enterocutaneous fistula is a rare complication that can arise many years after an umbilical hernia repair.
• Recurrence of the hernia may be a predisposing factor leading to mesh erosion into the underlying structures.
• Modification of repair technique and mesh type and location may aid in reducing the incidence of an enterocutaneous fistula.

## 1 Introduction

Ventral hernias are defined as non-inguinal, non-hiatal defects in the fascia of the abdominal wall. They can either be congenital or acquired [2]. Acquired ventral hernias are usually due to weakening or disruption of the fibro-muscular tissue of the abdominal wall that are affected by patient factors, technical factors, or a combination of both. The incidence of an incisional hernia is approximately 10–15% in patients with a prior abdominal incision, with midline incisions having the highest recurrence rates [3]. According to *Luijendijk et al.*, the 3-year cumulative risk of recurrence is higher with primary tissue repair accounting for 43% compared to 24% with mesh repair, which was thus recommended for all ventral hernias with a defect of more than one centimeter [4].

Multiple approaches are available to repair ventral hernias using a mesh placed at different sites; namely onlay, inlay, sublay, and underlay [5]. Nevertheless, several mesh-related complications have been reported, which can be associated with the type of mesh used, intraoperative findings, and surgical techniques used. Mesh related complications include infection, mesh migration, erosion into adjacent structures, enterocutaneous fistula formation, and hernia recurrence [6]. In this case report we present a 76 year old female with a history of an umbilical hernia repair with placement of a synthetic onlay mesh in 2009, presenting to a community hospital with an enterocutaneous fistula at the previous surgical site. This case was prepared and presented in accordance with the SCARE criteria and guidelines [7].

## 2 Presentation of case

We present a 76 years old obese female (BMI: 45 kg/m^2^) with a past medical history of type 2 diabetes, hypertension, and chronic renal insufficiency (baseline serum creatinine level: 127 mmol/l), who underwent an elective primary umbilical hernia repair with placement of a polypropylene onlay mesh ten years ago (2009). The patient described having an asymptomatic recurrence of her umbilical hernia for five years.

**Fig. 1:**
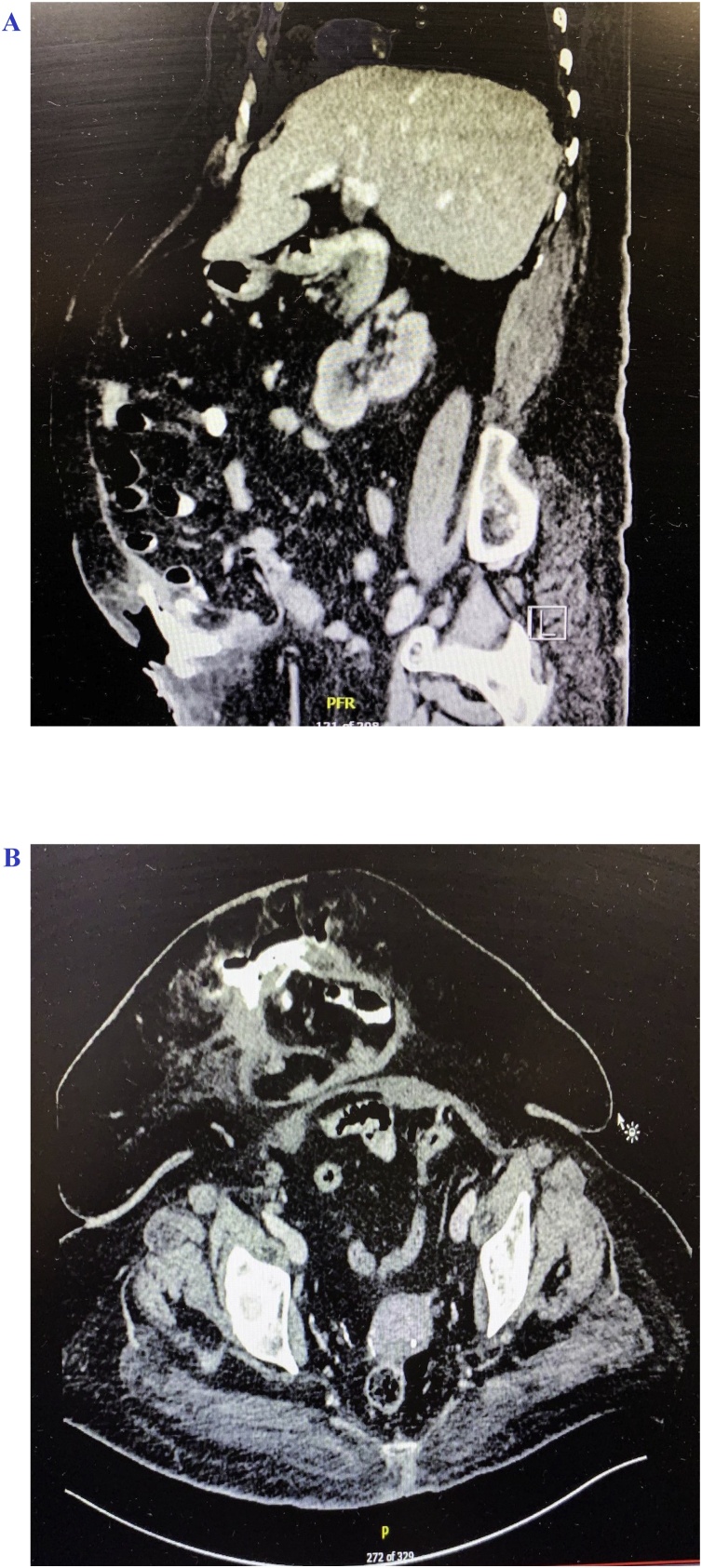
**A.** CT scan (sagittal images) with PO/IV contrast showing recurrent incisional hernia content with contrast pooling into subcutaneous tissue. **B.** CT scan (transverse images) with PO/IV contrast further delineating a recurrent incisional hernia with small bowel content and contrast pooling in the subcutaneous tissue.

She presented to the hospital with a spontaneously draining skin opening from the previous umbilical hernia incision site for 1-day duration. Upon physical examination, she was found to have an enterocutaneous fistula with skin induration and tenderness on palpation. Enteric content was draining from the skin opening. In addition, there was a large recurrent umbilical hernia under the fistula opening. The other areas of the abdomen were soft and lax on palpation, and she denied any symptoms of bowel obstruction. Her vital signs were within normal parameters. Her laboratory investigations revealed a white blood cell count of 32.9 × 10^9^/L and a hemoglobin level of 10 g/dL. A CT scan of the abdomen and pelvis with per oral (PO) and intravenous (IV) contrast was performed, which further confirmed the recurrence of the umbilical hernia along with the finding of oral contrast filling the small bowel loops. The oral contrast appeared to be pooling into the subcutaneous tissue overlying the hernia sac, delineating a communication between the small bowel lumen within the hernia sac and the overlying skin and subcutaneous tissue, thus forming an enterocutaneous fistula (Fig. 1A and B). No evidence of bowel obstruction was found.

We decided to take the patient to the operating room for an exploratory laparotomy and take down of the fistula with repair of the recurrent umbilical hernia.

**Fig. 2:**
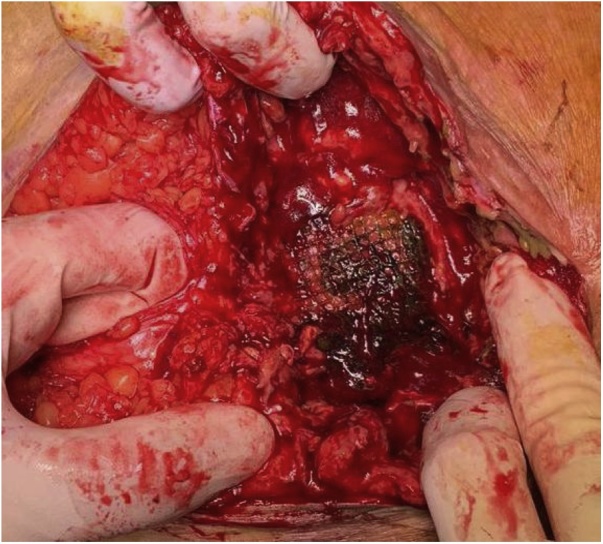
Surgical exploration revealing the site of enterocutaneous fistula with bile stained mesh overlying small bowel.

A midline laparotomy was performed. The onlay mesh was identified (Fig. 2). The surgery involved lysis of adhesions along with identification and reduction of the hernia contents, which included small bowel loops. No signs of bowel strangulation or obstruction were seen. The enterocutaneous fistula was identified, and the previously placed polypropylene mesh appeared to be adherent and eroding into the small bowel loop at the fistula site, located at the terminal ileum, 40 cm proximal to the ileocecal valve.

**Fig. 3:**
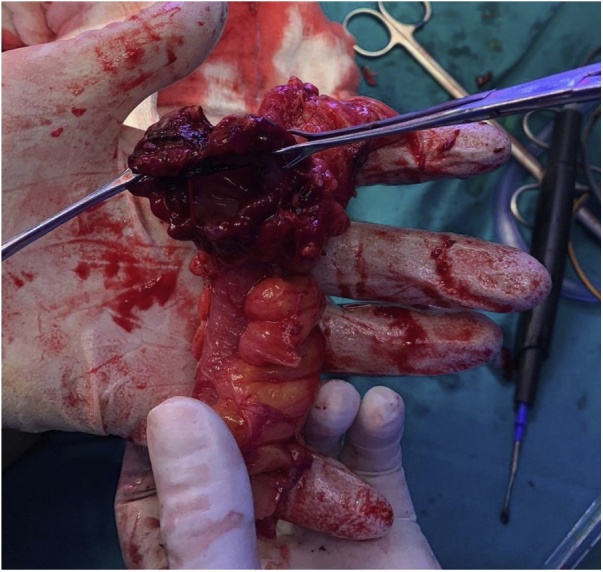
Specimen of a partial enterectomy with excised enterocutaneous fistula.

A segmental enterectomy was performed (Fig. 3), which involved removal of the small bowel fistula site as well as a stapled side-to-side small bowel anastomosis. The abdomen was irrigated and the abdominal fascia was closed primarily and the skin was left open for drainage. The patient had an uneventful recovery and was discharged home on the fourth post-operative day.

## 3 Discussion

Multiple studies have demonstrated a correlation between incisional hernias and numerous risk factors. These factors can be divided into technical and patient-related factors such as old age, obesity, smoking, malnutrition, connective tissue disorders and the use of immunosuppressive therapy. The technical factors include wound infection, the type and location of the mesh used, suboptimal fascial closure, and abdominal fascial dehiscence [1].

Regarding the techniques used in mesh repair, a meta-analysis done by *Holihan et al.* showed that sublay placement of a mesh had better outcome compared to onlay, inlay, and underlay repair [5]. The benefits of a sublay repair also include tissue integration with the posterior rectus sheath and anterior myofascial complex, as well as mesh protection from wound complications, contamination and intra-abdominal adhesions [5]. Another study suggested that the onlay and inlay mesh locations had the highest hernia recurrence rate (17% each). On the other hand, the sublay and underlay mesh locations were found to be superior in terms of lower hernia recurrence rates (5% and 7%, respectively) [8].

Another key aspect contributing to the weakening of the integrity a hernia repair is mesh migration. Mesh migration occurs mainly due to an inappropriately secured mesh, which can be displaced through the least resistant anatomical planes, as well as mesh erosion into nearby structures due to a foreign body reaction [9]. This process is gradual and may take several years to occur. As a consequence, these processes can in turn lead to infection and abscess formation. The literature further demonstrated that mesh migration and erosion could reach nearby organs such as the urinary bladder, cecum, as well as small bowel leading to a fistula formation [10].

With regards to our case, multiple factors may have contributed to the occurrence of the enterocutaneous fistula. The patient’s body habitus and obesity might have led to a chronic increase in intra-abdominal pressure that in turn weakened the primary ventral hernia repair, leading to a recurrent hernia. In addition, choosing an onlay mesh placement for this patient during the initial hernia repair was a sub-optimal choice in terms of the repair technique. Once the hernia repair was disrupted, the bare polypropylene mesh became exposed to the underlying small bowel content. The most probable pathophysiology is that the mesh became adherent and eroded into the small bowel over time with the subsequent enterocutaneous fistula formation.

Although ventral hernia repairs are associated with both short and long term complications, including the occurrence of enterocutaneous fistulae, it is important to advise on surgical and clinical modifications, as well as techniques to reduce or eliminate such complication.

One such modification is the position of the mesh, which might achieve a lower recurrence rate (sublay or underlay) [8].

Other aspects that might be improved include modification of patient risk factors such as reducing the weight prior to surgery. In obese patients, bariatric surgery might be a feasible option if indicated prior to considering a hernia repair [[11], [12], [13], [14]]. This aims to reduce the intra-abdominal pressure, which in turn might reduce the incidence of hernia recurrence and improve the outcome.

Newer products in the market might also aid in addressing the long term complication of an enterocutaneous fistula formation. These include the introduction of the newer bio-absorbable meshes. Our opinion focuses on the possibility of using a bio-absorbable mesh instead of a synthetic mesh during the initial repair. If a hernia recurrence was to happen after a long period of time, the body would have resorbed the mesh, and the hernia contents would not necessarily become in contact with a foreign body, which might lead to a possible fistula. The older generations of bio-absorbable meshes had a short life span and degraded within 3 months thus the recurrence rates were higher. Newer mesh subtypes have been developed, which show promising results regarding degradation time, which can be up to 18 months [15]. This might lead to less recurrence rates, however, more clinical trials are needed to compare the efficacy, safety, and cost effectiveness of the different meshes available.

## 4 Conclusion

Recurrence of a ventral hernia is a complication that might occur after a hernia repair with or without the use of mesh. In rare cases, the mesh may erode into the nearby structures, such as the small bowel, leading to an enterocutaneous fistula, which can present as a late complication after surgery. These complications could be reduced or avoided by adjusting technical and/or patient factors. After reviewing the literature, weight loss, sublay and underlay techniques, and bio-absorbable mesh may contribute to a better outcome.

## Funding

This study did not receive any financial support.

## Ethical approval

Case reports are exempted from ethical approval according to policies of The Ministry of Health in Kuwait.

## Consent

Written informed consent was obtained from the patient for publication of this case report and accompanying images.

## Author contribution

All authors contributed equally in gathering the data, images, performing a literature review and writing the paper.

Authors involved in this study are

Mohammed Alshamali

Sana Sallam

Dhari Alzaid

Joud Abdulraheem

Khaleel Mohammad

## Registration of research study

None.

## Guarantor

Khaleel Mohammad.

## Provenance and peer review

Not commissioned, externally peer-reviewed.

## Declaration of Competing Interest

There are no conflicts of interest to declare by authors.
